# Distinct basolateral amygdala excitatory inputs mediate the somatosensory and aversive-affective components of pain

**DOI:** 10.1016/j.jbc.2022.102207

**Published:** 2022-06-27

**Authors:** Xiaojing Meng, Lingxiao Yue, An Liu, Wenjuan Tao, Li Shi, Wan Zhao, Zhongmin Wu, Zhi Zhang, Liecheng Wang, Xulai Zhang, Wenjie Zhou

**Affiliations:** 1 Affiliated Psychological Hospital of Anhui Medical University, Hefei Fourth People’s Hospital, Anhui Mental Health Center, Hefei, China; 2 Department of Physiology, School of Basic Medical Sciences, Anhui Medical University, Hefei, China; 3 Department of Pathology, Anhui Medical College, Hefei, China; 4 Department of Otolaryngology, The First Affiliated Hospital of USTC, Division of Life Sciences and Medicine, University of Science and Technology of China, Hefei, Anhui, China; 5 Department of Anatomy, Medical College of Taizhou University, Taizhou, China; 6 Hefei National Laboratory for Physical Sciences at the Microscale, CAS Key laboratory of Brain Function and Disease, University of Science and Technology of China, Hefei, China

**Keywords:** pain, basolateral amygdala, circuit, insular cortex, mediodorsal thalamic nucleus, ACSF, artificial cerebrospinal fluid, AP, anterior-posterior, AP5, DL-2-amino-5-phosphonopentanoic acid, BLA, basolateral complex, BLAGlu, basolateral amygdala glutamatergic, CeA, central nucleus, CFA, complete Freund’s adjuvant, DNQX, 6,7-dinitroquinoxaline-2,3(1H,4H)-dione, DV, dorsoventral, EGFP, enhanced green fluorescent protein, Hepes, N-2-hydroxyethylpiperazine-N-2-ethanesulfonic acid, GABA, gamma-aminobutyric acid, IC, insular cortex, ICGlu, insular cortex glutamatergic, MDGlu, mediodorsal thalamic nucleus, ML, mediolateral, NMDG, N-methyl-d-glucosamine, RM, repeated-measures, RMP, resting membrane potential, RTPA, real-time place aversion, RTPP, real-time place preference, RV, rabies virus

## Abstract

Pain is a multidimensional perception that includes unpleasant somatosensory and affective experiences; however, the underlying neural circuits that mediate different components of pain remain elusive. Although hyperactivity of basolateral amygdala glutamatergic (BLA^Glu^) neurons is required for the somatosensory and emotional processing of pain, the precise excitatory inputs to BLA^Glu^ neurons and their roles in mediating different aspects of pain are unclear. Here, we identified two discrete glutamatergic neuronal circuits in male mice: a projection from the insular cortex glutamatergic (IC^Glu^) to BLA^Glu^ neurons, which modulates both the somatosensory and affective components of pain, and a projection from the mediodorsal thalamic nucleus (MD^Glu^) to BLA^Glu^ neurons, which modulates only the aversive-affective component of pain. Using whole-cell recording and fiber photometry, we found that neurons within the IC→BLA and MD→BLA pathways were activated in mice upon inflammatory pain induced by injection of complete Freund’s adjuvant (CFA) into their paws. Optical inhibition of the IC^Glu^→BLA pathway increased the nociceptive threshold and induced behavioral place preference in CFA mice. In contrast, optical inhibition of the MD^Glu^→BLA pathway did not affect the nociceptive threshold but still induced place preference in CFA mice. In normal mice, optical activation of the IC^Glu^→BLA pathway decreased the nociceptive threshold and induced place aversion, while optical activation of the MD^Glu^→BLA pathway only evoked aversion. Taken together, our results demonstrate that discrete IC^Glu^→BLA and MD^Glu^→BLA pathways are involved in modulating different components of pain, provide insights into its circuit basis, and better our understanding of pain perception.

Pain is an intricate feeling that involves sensory discrimination and emotional experience (1, 3, 2). Previous functional MRI studies on humans have revealed that both pain- and emotion-related nuclei were activated by painful stimuli (4, 5). Patients suffering from chronic pain frequently experience physical and psychological discomfort, such as anxiety and depression (6, 7, 8). However, the precise cell types and neural circuits mediating sensory discrimination and the emotional experience of pain remain largely unknown.

The amygdala is primarily composed of the basolateral complex (BLA), the central nucleus (CeA), and the intercalated cell clusters and plays a critical role in processing emotions and pain (9, 10, 11). The CeA is called the “nociceptive amygdala” and serves as the main output nucleus for the amygdala (11). Inhibition of the gamma-aminobutyric acid (GABA)ergic projection from the CeA to the lateral parabrachial nucleus is involved in the perception and pathogenesis of chronic pain (12). In contrast, BLA is considered as the input nucleus of the amygdala and receives inputs from external nuclei, such as the medial prefrontal cortex and anterior cingulate cortex. It is involved in processing pain and anxiety (13, 14). A previous study demonstrated that BLA lesions, rather than the CeA, inhibited chronic neuropathic pain (15) and abolished the aversion component of inflammatory pain (16, 17). Additionally, functional MRI studies in rodents and humans showed that neural hyperactivity and altered functional connectivity in the BLA are associated with the onset of chronic pain (18, 19). Activation of the excitatory projection from the BLA to the ventral hippocampus robustly increased anxiety-related behavior (20).

The BLA is primarily composed of glutamatergic neurons (21). In arthritic rats, the glutamatergic synaptic transmission in the BLA was enhanced (22), and inhibition of the enhanced excitatory synaptic transmission effectively increased the nociceptive threshold (an indicator of the somatosensory component of pain) and alleviated the aversive behaviors of pain (13, 23). In mice with chronic pain, the expression levels of TNF-α increased, which subsequently induced anxiety-like behavior by enhancing AMPA receptor–mediated glutamatergic excitatory synaptic transmission (24). In addition, the AMPA receptor antagonist tezampanel is used to treat pain (25, 26). Therefore, excitatory synaptic transmission in the BLA plays an important role in the development and maintenance of pain. However, the source of excitatory inputs to the BLA and their roles in mediating different aspects of pain require further investigation.

Combining viral tracing, optogenetics, fiber photometry, and electrophysiological recordings, we found that the IC^Glu^→BLA pathway processes both the somatosensory and aversive components of pain. In contrast, the MD^Glu^→BLA pathway was only involved in processing the aversive component of pain.

## Results

### Enhanced BLA^Glu^ neuronal activity in mice with inflammatory pain

**Figure 1:**
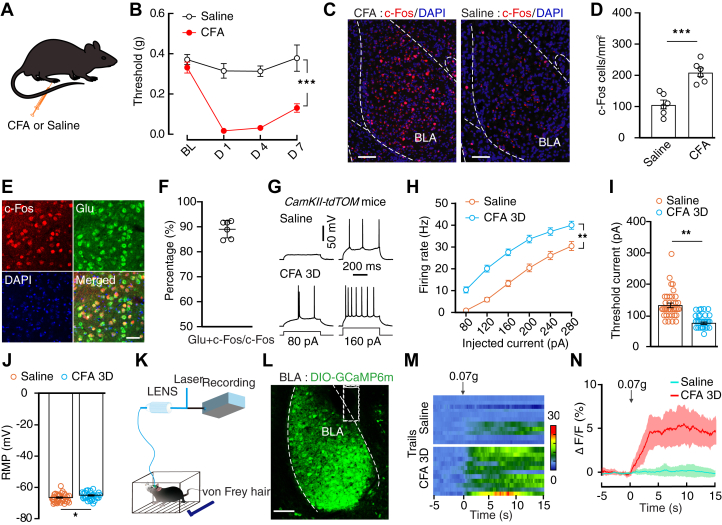
**Increased activity of BLA**^**Glu**^**neurons in inflammatory pain mice.***A*, schematic for CFA or saline injection. *B*, time course of mechanical nociceptive threshold based on the von Frey test (CFA, *n* = 7 mice; saline, *n* = 6 mice; time × group interaction, *F*_3,33_ = 17.62, *p* < 0.0001). *C* and *D,* representative images of c-Fos expression in the BLA from the indicated mice (C) and summarized data (D, *n* = 6 slices from three mice for each group; *t*_10_ = 5.27, *p* = 0.0004). The scale bar represents 100 μm in *C*. *E* and *F,* representative images of c-Fos^+^ neurons (*red*) that colocalized with glutamate immunofluorescence (*green*) in the BLA (E) and summarized data (F, *n* = 6 slices from three mice). The scale bar represents 50 μm in *E*. *G* and *H*, typical traces (G) and summarized data (H) of action potentials recorded in BLA^Glu^ neurons from different groups (saline, *n* = 36 cells; CFA 3D, *n* = 29 cells; time × group interaction, *F*_5,315_ = 3.445, *p* = 0.0048). *I* and *J,* summarized data of rheobase (*I*, *t*_63_ = 6.295, *p* < 0.0001) and RMP (J, *t*_63_ = 2.443, *p* = 0.0174) recorded in BLA^Glu^ neuron from saline and CFA 3D mice (saline, *n* = 36 cells from five mice; CFA 3D, *n* = 29 cells from four mice). *K*, schematic for fiber photometry recording in freely moving mice. *L*, representative image of AAV-DIO-GCaMP6m expression in the BLA of *CaMKII-Cre* mice. The scale bar represents 200 μm. *M* and *N,* the heatmaps (*M*) and mean data (*N*) of the Ca^2+^ signals that were recorded in BLA^Glu^ neurons from the indicated mice with subthreshold stimulation applied on hind paws (*n* = 5 mice). The colored bar on the *left* indicates ΔF/F (%). Significance was assessed by two-way repeated-measures (RM) ANOVA with *posthoc* comparison between groups B, H, and two-tailed unpaired Student’s *t* test (*D*), (*I*)*,* and (*J*). All data are presented as the mean ± SEM. ∗ *p* < 0.05, ∗∗ *p* < 0.01, ∗∗∗*p* < 0.001. BLA, basolateral complex; BLA^Glu^, basolateral amygdala glutamatergic; CFA, complete Freund’s adjuvant; RM, repeated-measures; RMP, resting membrane potential.

We established an inflammatory pain mouse model by administering complete Freund's adjuvant (CFA) into the left hind paw (Fig. 1, *A* and *B*). Three days later (3D), the expression levels of c-Fos, an immediate-to-early gene marker, significantly increased in the BLA in CFA-treated mice compared to the saline-treated control mice (Saline 3D; Fig. 1, *C* and *D*). Meanwhile, immunostaining showed that the c-Fos^+^ neurons were predominantly colocalized with glutamate immunofluorescence signals (Fig. 1, *E* and *F*).

Moreover, we crossed *CaMKII-Cre* mice that express Cre recombinase in the excitatory neurons with Ai14 reporter mice to produce transgenic mice (*CaMKII-tdT*) with tdTomato expression in glutamatergic neurons for visualized recording. Whole-cell recordings of visualized basolateral amygdala glutamatergic (BLA^Glu^) neurons showed an increase in the spike number and resting membrane potential (RMP) and decreased rheobase in CFA 3D mice compared to those recorded in the control group (Fig. 1, *G*–*J*).

An adeno-associated virus expressing the Cre-dependent fluorescent Ca^2+^ indicator GCaMP6m (AAV-DIO-GCaMP6m) was then injected into the BLA of *CaMKII-Cre* mice to monitor the Ca^2+^ transient frequency of BLA^Glu^ neurons in freely moving mice (Fig. 1, *K* and *L*). Application of subthreshold stimulation (0.07-g von Frey filament) onto the hind paw rapidly increased the Ca^2+^ transient frequency of BLA^Glu^ neurons in CFA 3D but not in saline-treated mice (Fig. 1M, N).

**Figure 2:**
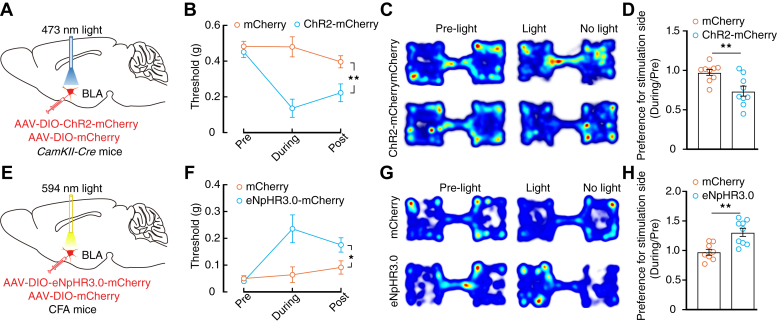
**Essential role of BLA**^**Glu**^**neurons in inflammatory pain-induced mechanical allodynia and aversion.***A*, schematic for optical activation of BLA^Glu^ neurons. *B*, mechanical nociceptive threshold of normal *CaMKII-Cre* mice following photostimulation of BLA^Glu^ neurons from the indicated group (*n* = 7 mice in each group; time × group interaction, *F*_2,24_ = 9.148, *p* = 0.0011). *C*, heatmaps showing the locations of normal mice in the RTPA test before and during photostimulation of BLA^Glu^ neurons from the indicated mice (*C*) and summarized data (*D*, *n* =9 mice in each group; *t*_16_ = 3.22, *p* = 0.0054). *E*, schematic of viral injection for optical inhibition of BLA^Glu^ neurons. *F*, mechanical nociceptive threshold of *CaMKII-Cre* mice treated with CFA for 3 d following optical inhibition of BLA^Glu^ neurons (*n* = 7 mice in each group; time × group interaction, *F*_2,24_ = 4.237, *p* = 0.0265). *G*, heatmaps showing the locations of CFA 3D mice in the RTPP test before and during photostimulation of BLA^Glu^ neurons from the indicated group (*G*) and summarized data (*H*, mCherry, *n* = 8 mice; eNpHR3.0-mCherry, *n* = 9 mice; *t*_15_ = 3.814, *p* = 0.0017). Significance was assessed by two-way RM ANOVA with *posthoc* comparison between groups in (*B*), (*F*), and two-tailed unpaired Student’s *t* test in (*D*) and (*H*). The data are expressed as the mean ± SEM. ∗*p* < 0.05; ∗∗*p* < 0.01. BLA^Glu^, basolateral amygdala glutamatergic; CFA, complete Freund’s adjuvant; RTPA, real-time place aversion; RM, repeated-measures; RTPP, real-time place preference.

For artificial activation of BLA^Glu^ neurons, an adeno-associated virus expressing Cre-dependent channelrhodopsin-2 (AAV-DIO-ChR2-mCherry) was injected into the BLA of *CaMKII-Cre* mice (Fig. 2*A*). Optical activation of BLA^Glu^ neurons decreased the nociceptive threshold of normal mice and induced real-time place aversion (RTPA) for the light delivery side (Fig. 2, *B*–*D*). Furthermore, an adeno-associated virus expressing Cre-dependent eNpHR3.0-EYFP (AAV-DIO-eNpHR3.0-EYFP) was injected into the BLA of *CaMKII-Cre* mice to selectively inhibit BLA^Glu^ neurons (Fig. 2*E*). Optical inhibition of the BLA^Glu^ neurons increased the mechanical nociceptive threshold and induced real-time place preference (RTPP) for the stimulation paired side in CFA mice (Fig. 2, *F*–*H*). These data suggest that the hyperactivity of BLA^Glu^ neurons was involved in processing the somatosensory and aversive-affective components of pain.

### Inflammatory pain increases excitatory synaptic transmission in the BLA

**Figure 3:**
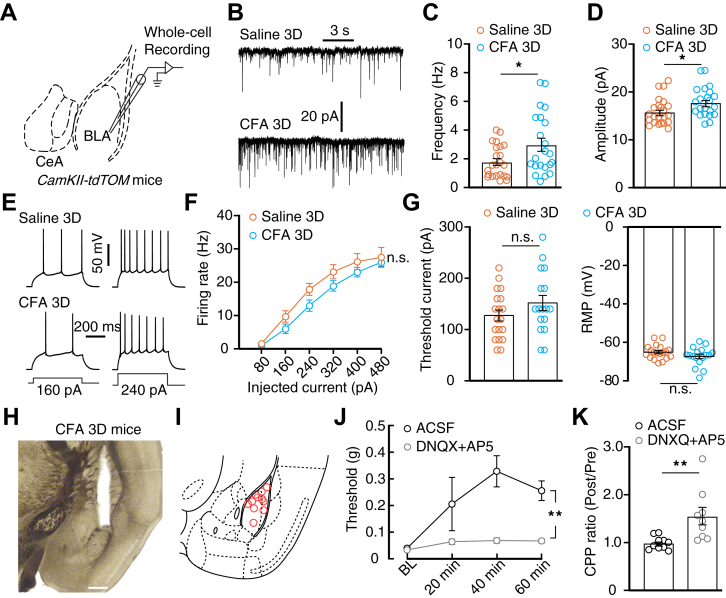
**Enhanced excitatory synaptic transmission in the BLA was required for neural hyperactivity and maintenance of pain.***A*, schematic for whole-cell recording in acute brain slice. *B–D*, typical traces (*B*) of the mEPSC recorded in BLA^Glu^ neurons from the indicated group and summarized data of the frequency (*C*, saline 3D, *n* = 23 cells from four mice; CFA 3D, *n* = 22 cells from four mice; *t*_43_ = 2.383, *p* = 0.0217) and amplitude (*D*, *t*_43_ = 2.237, *p* = 0.0305). *E* and *F*, typical action potential traces (*E*) and summarized data (*F*, time × group interaction, *F*_5,170_ = 1.121, *p* = 0.351) recorded in BLA^Glu^ neurons from the indicated group after perfusion of DNQX and AP5 (saline 3D, *n* = 19 cells from four mice; CFA 3D, *n* = 17 cells from three mice). *G*, summarized data of the rheobase (left, *t*_34_ = 1.346, *p* = 0.1872) and RMP (right, *t*_34_ = 1.566, *p* = 0.1267) recorded in BLA^Glu^ neurons from the indicated group after perfusion of DNQX and AP5. *H* and *I*, representative image (*H*) and drawing of cannula implantation sites (*I*). The scale bar represents 200 μm. *J* and *K*, mechanical nociceptive threshold (*J*, *n* = 7 mice in each group; time × group interaction, *F*_3,36_ = 2.661, *p* = 0.0627) and summarized data of CPP ratio (K, ACSF, *n* = 10 mice; DNQX+AP5, *n* = 9 mice; *t*_17_ = 3.176, *p* = 0.0055) of CFA 3D mice following the BLA microinjection of ACSF or DNQX+AP5. Significance was assessed by a two-tailed unpaired Student’s *t* test in (*C*), (*D*), (*G*), (*K*), and two-way RM ANOVA with *posthoc* comparison between groups in (*F*) and (*J*). The data are expressed as the mean ± SEM. ∗*p* < 0.05; ∗∗*p* < 0.01; n.s., no significance. ACSF, artificial cerebrospinal fluid; AP5, DL-2-amino-5-phosphonopentanoic acid; BLA, basolateral complex; BLA^Glu^, basolateral amygdala glutamatergic; CFA, complete Freund’s adjuvant; CPP, conditioned place preference; DNQX, 6,7-dinitroquinoxaline-2,3(1H,4H)-dione; mEPSC, miniature excitatory postsynaptic current; RM, repeated-measures; RMP, resting membrane potential.

Increased excitatory synaptic transmission in the BLA is important for pain sensation (27). Therefore, we evaluated how increased excitatory synaptic transmission impacts pain-induced hyperactivity of BLA^Glu^ neurons. First, whole-cell recording of BLA^Glu^ neurons showed that the frequency and amplitude of the miniature excitatory postsynaptic current were enhanced in CFA 3D mice compared to the controls (Fig. 3, *A*–*D*). Excitatory synaptic transmission was then blocked by the perfusion of 6,7-dinitroquinoxaline-2,3(1H,4H)-dione (DNQX) and DL-2-amino-5-phosphonopentanoic acid (AP5). After blocking the excitatory synaptic transmission, the differences in firing rate, RMP, and rheobase between CFA 3D and control mice were eliminated (Fig. 3, *E*–*G*). Furthermore, microinjection of DNQX and AP5 into the BLA significantly increased the nociceptive threshold of CFA 3D mice and induced a conditioned place preference for the drug-paired side (Fig. 3, *H*–*K*).

**Figure 4:**
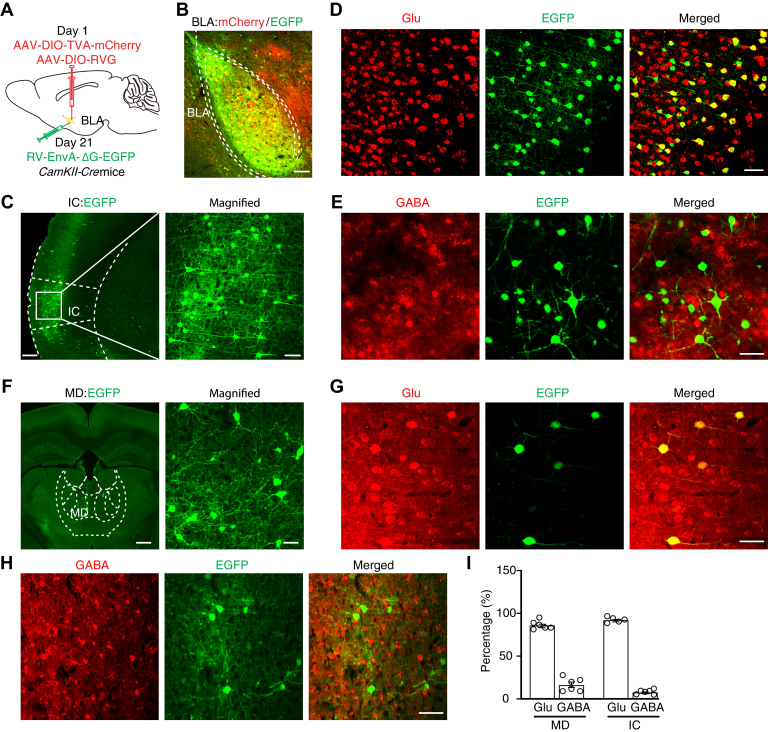
**Inputs to BLA**^**Glu**^**neurons.***A*, schematic for retrograde trans-monosynaptic tracing. *B*, representative image of the viral expression within the BLA of *CaMKII-Cre* mice. Starter cells (*yellow*) coexpressing AAV-DIO-TVA–mCherry (*red*), AAV-DIO–RVG, and rabies RV-EnvA-ΔG–EGFP (*green*). The scale bar represents 200 μm. *C*, *D*, and *E*, representative images of the EGFP-labeled neurons within the IC (*C*) colocalized with Glu immunofluorescence (*D*) but few with GABA immunofluorescence (*E*). The scale bar represents 200 μm (*left*) and 100 μm (*right*) in (*C*). The scale bar represents 50 μm in (*D*) and (*E*). *F*, *G*, and *H*, representative images of the EGFP-labeled neurons within the MD (*F*) colocalized with Glu immunofluorescence (*G*) but few with GABA immunofluorescence (*H*). The scale bar represents 200 μm (*left*) and 100 μm (*right*) in (*F*). The scale bar represents 50 μm in (*G*) and (*H*). *I*, summarized data of EGFP-positive neurons in the MD and IC that colocalized with glutamate or GABA immunofluorescence (n = 5 or 6 slices from three mice). Each dot represents one slice. BLA, basolateral complex; BLA^Glu^, basolateral amygdala glutamatergic; IC, insular cortex; RV, rabies virus.

Based on the above data, we then explored the excitatory inputs to BLA^Glu^ neurons. The Cre-dependent helper viruses (AAV-EF1α-DIO-TVA-mCherry and AAV-EF1α-DIO-RVG) were injected into the BLA of *CaMKII-Cre* mice. After 3 weeks, the rabies virus (RV, EnvA-pseudotyped RV-ΔG-EGFP) was injected into the same site (Fig. 4, *A* and *B*). Intense enhanced green fluorescent protein (EGFP)-labeled neurons were observed in the piriform cortex, ectorhinal cortex, posterior thalamic nuclear group, parafascicular thalamic nucleus, auditory cortex, insular cortex (IC), and MD (Figs. 4, *C* and *F*, and S1). Because the IC and MD are known to modulate different components of pain, we subsequently focused on the IC→BLA and MD→BLA circuits. Immunofluorescence staining showed that the EGFP^+^ neurons in the IC were mainly colocalized with glutamate-specific antibodies but not GABA antibodies (Fig. 4, *D* and *E*). Similar immunostaining results were observed in the MD (Fig. 4, *G*–*I*).

### Essential role of the IC^Glu^→BLA pathway in CFA-induced mechanical allodynia and RTPA

**Figure 5:**
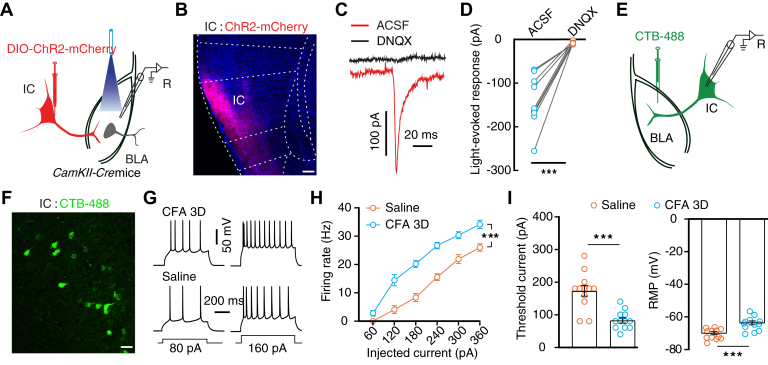
**Inflammatory pain activated BLA-projecting IC neurons.***A*, schematic for viral injection and whole-cell recording in acute brain slices. *B*, representative image of AAV-DIO-ChR2-mCherry expression in the IC of *CaMKII-Cre* mice. The scale bar represents 100 μm. *C* and *D*, typical traces (*C*) and summarized data (*D*, n = 9 cells from 3 mice, t8 = 6.596, p = 0.0002) of light-evoked postsynaptic current that was recorded from BLA neurons following optical activation of IC^Glu^ terminals without or with DNQX. *E*, schematic of CTB-488 injection and whole-cell recording in an acute brain slice. *F*, representative image of CTB-488-labeled neurons in the IC. The scale bar represents 50 μm. *G* and *H*, typical action potential traces (*G*) and summarized data (*H*, *n* = 13 cells in each group; time × group interaction, *F*_5,110_ = 6.369, *p* < 0.0001) recorded in BLA-projecting IC neurons from the indicated group. *I*, summarized data of rheobase (left, *t*_22_ = 4.922, *p* < 0.0001) and RMP (right, *t*_22_ = 4.161, *p* = 0.0004) recorded from BLA-projecting IC neurons from the indicated group. Significance was assessed by a two-tailed paired Student’s *t* test in (*D*), two-way RM ANOVA with *posthoc* comparison between groups in (*H*), and a two-tailed unpaired Student’s *t* test in (*I*). The data are expressed as the mean ± SEM. ∗∗∗*p* < 0.001. BLA, basolateral complex; DNQX, 6,7-dinitroquinoxaline-2,3(1H,4H)-dione; IC, insular cortex; IC^Glu^, insular cortex glutamatergic; RM, repeated-measures; RMP, resting membrane potential.

We then examined the roles of the IC^Glu^→BLA and MD^Glu^→BLA pathways in pain processing. To characterize the role of the IC^Glu^→BLA pathway, we delivered AAV-DIO-ChR2-mCherry into the IC of *CaMKII-Cre* mice and performed whole-cell recording on BLA neurons (Fig. 5, *A* and *B*). Brief optical stimulation of ChR2-containing IC^Glu^ terminals elicited excitatory postsynaptic currents in BLA neurons, which could be blocked by administering the antagonist DNQX (Fig. 5, *C* and *D*). We then recorded the BLA-projecting IC neurons labeled by the retrograde tracer CTB-488 injected in the BLA (Fig. 5, *E* and *F*). The whole-cell recording showed that the firing rate and RMP of the BLA-projecting IC neurons increased and rheobase decreased in CFA 3D mice compared to those in the control mice (Fig. 5,*G*–*I*).

**Figure 6:**
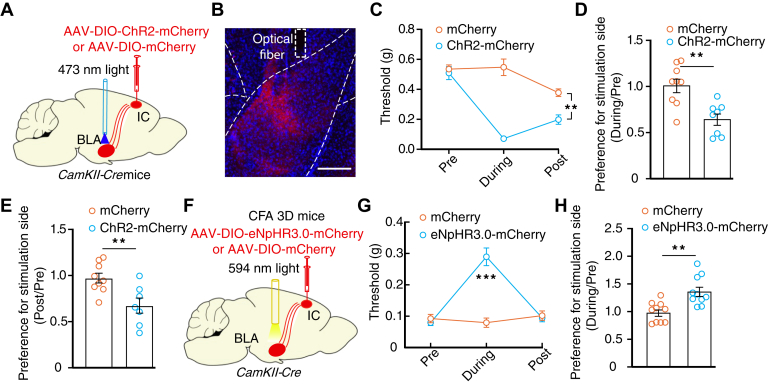
**Essential role of the IC**^**Glu**^**→BLA pathway in pain-induced allodynia and RTPA.***A*, schematic for optical activation of the IC^Glu^→BLA pathway in *CaMKII-Cre* mice. *B,* representative image of optical fiber implantation site. The scale bar represents 100 μm. *C* and *D,* nociceptive threshold (C, time × group interaction, *F*_2,30_ = 21.07, *p* < 0.0001) and RTPA ratio (D, *t*_15_ = 3.796, *p* = 0.0018) of normal *CaMKII-Cre* mice following optical activation of the IC^Glu^→BLA pathway (mCherry, *n* = 9 mice; ChR2-mCherry, *n* = 8 mice). *E*, summarized data of conditioned place aversion ratio following optical activation of the IC^Glu^→BLA pathway (mCherry, *n* = 9 mice; ChR2-mCherry, *n* = 7 mice; *t*_14_ = 3.180, *p* = 0.0067). *F,* schematic of viral injection for optical activation of the IC^Glu^→BLA pathway in *CaMKII-Cre* mice treated with CFA for 3 days *G* and *H,* nociceptive threshold (G, mCherry, *n* = 10 mice in each group; time × group interaction, *F*_2,36_ = 44.72, *p* < 0.0001) and place preference ratio (*H*, *t*_18_ = 3.761, *p* = 0.0014) of CFA 3D mice following optical inhibition of the IC^Glu^→BLA pathway. Significance was assessed by two-way RM ANOVA with *posthoc* comparison between groups in (*C*) and (*G*) and two-tailed unpaired Student’s *t* test in (*D*), (*E*), and (*H*). The data are expressed as the mean ± SEM. ∗∗*p* < 0.01; ∗∗∗*p* < 0.001. BLA, basolateral complex; CFA, complete Freund’s adjuvant; IC^Glu^, insular cortex glutamatergic; RM, repeated-measures; RTPA, real-time place aversion.

We then injected AAV-DIO-ChR2-mCherry into the IC and implanted an optical fiber that targeted the BLA to selectively activate the IC^Glu^→BLA pathway (Fig. 6, *A* and *B*). After 4 weeks, optical activation of ChR2-containing fibers in the BLA decreased the nociceptive threshold of normal mice (Fig. 6*C*). Additionally, this manipulation induced RTPA and conditioned place aversion for the light delivery side (Fig. 6, *D* and *E*). In contrast, optical inhibition of eNpHR-containing IC^Glu^ terminals in the BLA increased the nociceptive threshold of CFA 3D mice and induced RTPP (Fig. 6, *F*–*H*).

### MD^Glu^→BLA pathway activation induced aversion rather than allodynia

**Figure 7:**
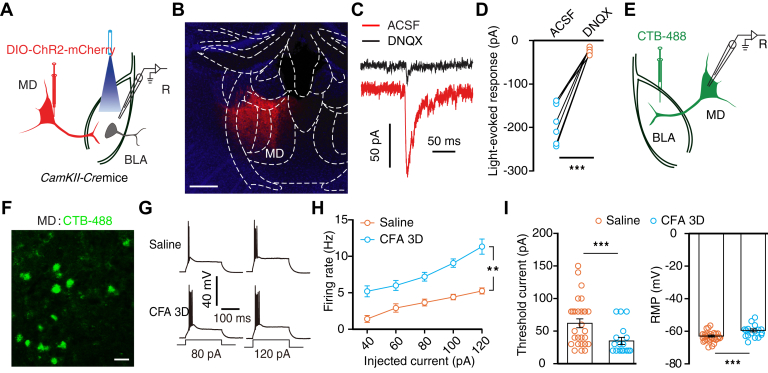
**Inflammatory pain activated BLA-projecting MD neurons.***A*, schematic for viral injection and whole-cell recording in brain slice. *B*, representative image of AAV-DIO-ChR2-mCherry expression in the MD of *CaMKII-Cre* mice. The scale bar represents 100 μm. *C* and *D,* typical traces (*C*) and summarized data (*D*, *n* = 6 cells from three mice, *t*_5_ = 9.25, *p* < 0.0001) of light-evoked postsynaptic current recorded from BLA neurons following optical activation of MD^Glu^ terminals without or with DNQX. *E,* schematic of CTB-488 injection and whole-cell recording in acute brain slice. *F,* representative image of CTB-488–labeled neurons in the MD. The scale bar represents 50 μm. *G* and *H,* typical action potential traces (*G*) and summarized data (*H*, saline, *n* = 24 cells from five mice; CFA 3D, *n* = 15 cells from four mice; time × group interaction, *F*_4,148_ = 3.841, *p* = 0.0053) recorded in BLA-projecting MD neurons from the indicated group. *I,* summarized data of rheobase (*left*, *t*_37_ = 3.23, *p* = 0.0026) and RMP (*right*, *t*_37_ = 4.828, *p* < 0.0001) recorded in BLA-projecting MD neurons from the indicated group. Significance was assessed by a two-tailed paired Student’s *t* test in (*D*), two-way RM ANOVA with *posthoc* comparison between groups in (*H*), two-tailed unpaired Student’s *t* test in (*I*). The data are expressed as the mean ± SEM. ∗∗*p* < 0.01; ∗∗∗*p* < 0.001. BLA, basolateral complex; CFA, complete Freund’s adjuvant; DNQX, 6,7-dinitroquinoxaline-2,3(1H,4H)-dione; MD, mediodorsal thalamic nucleus; RM, repeated-measures; RMP, resting membrane potential.

We then evaluated the role of the MD^Glu^→BLA pathway in pain processing. The light-evoked excitatory postsynaptic currents, which could be blocked by administering DNQX, were reliably recorded from the BLA neurons following optical stimulation of ChR2-containing MD^Glu^ terminals (Fig. 7, *A*–*D*). Additionally, electrophysiological recordings showed that the firing rate and RMP of the BLA-projecting MD neurons, labeled by the retrograde tracer CTB-488 injected in the BLA, were higher in CFA 3D mice compared to the controls (Fig. 7, *E*–*H*). However, rheobase was decreased (Fig. 7*I*).

**Figure 8:**
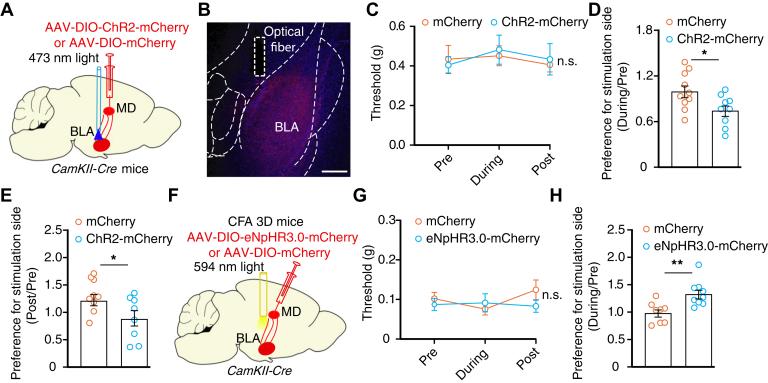
**MD**^**Glu**^**→BLA pathway involved in pain-induced aversion rather than allodynia.***A*, schematic for optical activation of MD^Glu^→BLA pathway. *B*, representative image of optical fiber implantation site. The scale bar represents 100 μm. *C* and *D,* nociceptive threshold (C, time × group interaction, *F*_2,34_ = 0.4036, *p* = 0.6711) and RTPA ratio (*D*, *t*_17_ = 2.43, *p* = 0.0265) following optical activation of the MD^Glu^→BLA pathway in normal *CaMKII-Cre* mice (mCherry, *n* = 10 mice; ChR2-mCherry, *n* = 9 mice). *E,* summarized data of conditioned place aversion ratio following optical activation of the MD^Glu^→BLA pathway (mCherry, *n* = 9 mice; ChR2-mCherry, *n* = 7 mice; *t*_14_ = 3.180, *p* = 0.0067). *F,* schematic of viral injection for optical inhibition of the MD^Glu^→BLA pathway in *CaMKII-Cre* mice treated with CFA for 3 days. *G* and *H*, nociceptive threshold (G, *F*_2,30_ = 0.8479, *p* = 0.4383), and place preference ratio (H, *t*_15_ = 3.299, *p* = 0.0049) of CFA 3D *CaMKII-Cre* mice following optical inhibition of the MD^Glu^→BLA pathway (mCherry, *n* = 8 mice; eNpHR3.0-mCherry, n = 9 mice). Significance was assessed by two-way RM ANOVA with *posthoc* comparison between groups in (*C*), (*G*)*,* and two-tailed unpaired Student’s *t* test in (*D*), (*E*), and (*H*). The data are expressed as the mean ± SEM. ∗*p* < 0.05; ∗∗*p* < 0.01; n.s., no significance. BLA, basolateral complex; CFA, complete Freund’s adjuvant; MD^Glu^, mediodorsal thalamic nucleus; RM, repeated-measures; RTPA, real-time place aversion.

Optical activation of the MD^Glu^→BLA pathway did not affect the mechanical nociceptive threshold of normal mice (Fig. 8, *A*–*C*) but induced RTPA and conditioned place aversion on the light delivery side (Fig. 8, *D* and *E*). In addition, no overt change was observed in the nociceptive threshold (Fig. 8, *F* and *G*), but RTPP was induced in the CFA 3D mice following optical inhibition of this pathway (Fig. 8*H*). Together, we identified two discrete glutamatergic neuronal circuits that mediate, at least in part, the somatosensory and aversion components of pain.

## Discussion

In this study, we found that the enhanced excitatory synaptic transmission in the BLA is essential for maintaining the hyperactivity of BLA^Glu^ neurons and inflammation-induced pain hypersensitivity. Furthermore, through viral tracing and optogenetics, we determined that the IC^Glu^→BLA pathway modulates both the somatosensory and aversive components of pain, but the MD^Glu^→BLA pathway is only involved in modulating the aversive component of pain.

Previous field studies have primarily focused on the maladaptation caused by pain in the spinal cord and brain and explored promising targets for treating chronic pain (28, 29). However, pain is an intricate and emotional experience, and the underlying neural circuits remain unclear. Previous *in vivo* calcium imaging studies reported that the activation of BLA pyramidal neurons is required for the negative affective components of pain (17). Inhibiting excitatory synaptic transmission by microinjection of the metabotropic glutamate receptor 1 antagonist into the BLA produced analgesic effects (30). However, the underlying precise neural circuits remain unexplored.

The essential role of the increased excitatory synaptic transmission in the BLA in pain processing is known, but the precise excitatory inputs remain elusive. BLA receives polymodal information from the cortex and thalamus. The IC is a multisensory integration region that processes pain perception, memory, empathy, and emotion (31, 32, 33, 34). Direct electrical stimulation of the IC enhanced pain perception, and chemical inhibition of the IC produced analgesia (35, 36). Patients with strokes, including those affecting the IC, suffer from a lack of pain and emotional response to painful stimuli (37, 38). In addition, there are massive connections between the IC and the amygdala, but the functional role of these connections remains elusive. Using viral tracing, optogenetics, and whole-cell recording, we revealed that the IC^Glu^→BLA pathway was required for the sensory discriminative and aversive-affective components of pain. The multiple effects of the IC^Glu^→BLA pathway in pain processing are likely caused by different subregions of the IC because the posterior IC is preferentially involved in processing somatosensory features of pain, while the anterior IC preferentially mediates the affective component (39, 40, 41, 42).

Previous studies in humans revealed abnormal activity of the MD in patients with chronic pain (43, 44). In rats, both spontaneous firing rates and responses to noxious stimuli of MD neurons were increased following spinal cord injuries (45). A previous study showed that the directly ascending projections from the spinal cord to the MD are required for painful stimuli-induced coping behaviors (46). Moreover, the MD is heavily connected to cortical areas, such as medial prefrontal cortex, IC, and anterior cingulate cortex, which function in pain-related aversion (47, 48, 49, 50). Therefore, MD is one of the main components of the medial pain pathway that is involved in the affective dimension of pain (51). Our optogenetic experiments revealed that activation of the MD^Glu^→BLA pathway primarily mediated the aversive dimension of pain rather than the somatosensory discrimination of pain.

This study characterized the IC^Glu^→BLA and MD^Glu^→BLA pathways through which the somatosensory and aversion components of pain perception are generated. Our results provide a preliminary description of cortical and thalamic neural circuits for coding the somatosensory and aversive-affective components of pain.

## Experimental procedures

### Animals

Male mice (8–12-week-old), including C57BL/6J, *CaMKII-Cre*, and Ai14 (RCL-TDT), were purchased from the Charles River or Jackson Laboratories. Mice (3–5/cage) were raised at room temperature (23–25 °C), under a 12-h light/dark cycle, and allowed free access to water and food (standard mouse chow). All experiments were approved by the Animal Care and Use Committee of the University of Science and Technology of China.

### Inflammatory pain mouse model

Persistent inflammatory pain was induced by administering CFA (20 μl; Sigma-Aldrich) into the plantar vein of the left hind paw using an insulin syringe (BD Corp.) under deep anesthesia with isoflurane. The control mice were administered normal saline (0.9% NaCl).

### Von Frey filament test

Von Frey test was used to measure the mechanical nociceptive threshold, as reported previously (52). Before the experiment, mice were placed in the testing room for at least 30 min for habituation. Then, the mice were placed separately in a transparent plastic chamber on a wire mesh and the calibrated von Frey filaments were applied to the surface of the hind paw with subsequently increasing force. The mechanical response threshold was determined as the minimal force filament that induced mice to present licking, biting, or sudden withdrawal of the hind paw. For optogenetic manipulations, the von Frey filaments were applied 1 min after the light was delivered, and the withdrawal threshold was calculated as the average of five applications.

### Virus injection

After deep anesthesia with isoflurane, mice were fixed in a stereotactic frame (RWD) and a heating pad was used to maintain the core body temperature at 36 °C. The virus used for tracing and optogenetic manipulation was delivered into the target regions using an infusion pump (micro 4, WPI) at a rate of 25 nl/min. The coordinates were defined as dorsoventral (DV) from the brain surface, anterior-posterior (AP) from the bregma, and mediolateral (ML) from the midline (in mm). Since the CFA was injected into the left hind paws and the nociceptive signals from the hind paws were transmitted to the contralateral brain, the virus was unilaterally injected in the right hemispheres.

For retrograde monosynaptic tracing, helper viruses that contained rAAV-Ef1α-DIO-RVG-WPRE-pA (AAV-DIO-RVG, AAV2/9, 2 × 10^12^ viral genome, vg/ml) and rAAV-Ef1α-DIO-mCherry-2a-TVA-WPRE-pA (AAV-DIO-TVA-mCherry, AAV2/9, 2 × 10^12^ vg/ml; 1:1, 200 nl) were coinjected into the BLA (AP, −1.25 mm; ML, −3.05 mm; DV, −4.05 mm) of *CaMKII-Cre* mice. Three weeks later, the rabies virus RV-ENVA-ΔG-EGFP (2 × 10^8^ IFU/ml, 250 nl) was delivered to the same site. Ten days after the RV injection, mice were deeply anesthetized and transcardially perfused. Finally, the brain was sliced and prepared for detecting the EGFP signal or performing immunofluorescence staining with glutamate-specific or GABA-specific antibodies.

For anterograde tracing, the Cre-dependent virus rAAV-Ef1α-DIO-hChR2(H134 R)-mCherry-WPRE-pA (AAV-DIO-ChR2-mCherry, AAV2/9, 1.63 × 10^13^ vg/ml, 200 nl) was delivered into the IC and MD of *CaMKII-Cre* mice. After three weeks, the mice were transcardially perfused and the mCherry signals were detected in the whole brain.

For optical activation or inhibition of BLA^Glu^ neurons, the Cre-dependent AAV-DIO-ChR2-mCherry or rAAV-Ef1α-DIO-eNpHR3.0-mCherry-WPRE-pA (AAV-DIO-eNpHR3.0-mCherry, AAV2/9, 1.18 × 10^13^ vg/ml) was infused into the BLA of *CaMKII-Cre* mice; an optical fiber (diameter, 200 μm; Newdoon) was implanted into the same site and secured to the skull with dental cement. For optical activation or inhibition of IC^Glu^→BLA pathway, the Cre-dependent AAV-DIO-ChR2-mCherry or AAV-DIO-eNpHR3.0-mCherry was infused into the IC (AP, +1.6 mm; ML, −3.05 mm; DV, −2.0 mm with a 6° angle) of *CaMKII-Cre* mice, and an optical fiber was implanted toward the BLA. For optical activation or inhibition of MD^Glu^→BLA pathway, AAV-DIO-ChR2-mCherry or AAV-DIO-eNpHR3.0-mCherry was infused into the MD (AP, −1.58 mm; ML, −0.5 mm; DV, −3.10 mm with a 6° angle) of *CaMKII-Cre* mice, and an optical fiber was implanted toward the BLA. All viruses were packaged by BrainVTA.

### Fiber photometry recording in freely moving mice

The fiber photometry recording was conducted using a commercial fiber photometry recording system (Inper), as previously reported (53, 54). First, the Cre-dependent virus rAAV-Ef1α-DIO-Gcamp6m-WPRE-pA (AAV-DIO-Gcam6m, AAV2/9, 2.53 × 10^12^ vg/ml, 200 nl) was injected into the BLA of *CaMKII-Cre* mice and the optical fibers (0.37 NA) were implanted using dental acrylic. After the virus injection, the mice were housed individually for 2 weeks. For fluorescence recording, a laser beam from a laser tube (488 nm) was reflected by a dichroic mirror, focused by a 10 × lens (NA = 0.3), and connected to an optical commutator (Doric Lenses). An optical fiber was used to guide the signal between the commutator and the implanted optical fiber. A bandpass filter was applied to the GCaMP fluorescence (MF525-39; Thorlabs) and collected by a photomultiplier tube (R3896, Hamamatsu Photonics). An amplifier (C7319, Hamamatsu Photonics) was used to convert the photomultiplier tube current output to voltage signals, which were passed through a low-pass filter (40 Hz cut-off; Brownlee 440). The analog voltage signals were digitalized at 500 Hz and recorded by a Power 1401 digitizer and Spike2 software (https://ced.co.uk/products/spkovin). We derived the values of fluorescence change (Δ*F/F*) by calculating Δ*F*/*F0* = *F*(*t*) − *F*0(*t*)/*F*0(*t*) using software written by the Inper company.

### Immunofluorescence

The mice were anesthetized with isoflurane and intraperitoneal pentobarbital sodium (50 mg/kg, i.p.). Under deep anesthesia, the mice were transcardially perfused with ice-cold 0.9% saline and 4% (w/v) paraformaldehyde. Then, the brain was removed and postfixed in 4% paraformaldehyde at 4 °C for at least 24 h. After cryoprotection with 30% (w/v) sucrose, coronal sections were cut using a cryostat (Leica CM1860) at 40 μm thickness. After washing with PBS three times (5 min each), the brain slices were incubated with 5% (v/v) donkey serum in PBS containing 0.3% Triton X-100 for 1 h at room temperature and incubated for 24 to 48 h at 4 °C with the following primary antibodies: anti-glutamate (1:500, rabbit, Sigma-Aldrich), anti-GABA (1:500, rabbit, Sigma-Aldrich), and anti-c-Fos (1:500, goat, Santa Cruz Biotechnology). Finally, the sections were incubated with corresponding fluorophore-conjugated secondary antibodies (1:500, Invitrogen) for 2 h at room temperature and photographed using a Zeiss LSM880 after washing with PBS three times.

### *In vivo* optogenetic manipulations

The optogenetic manipulations were performed as previously reported. In brief, mice were anesthetized with isoflurane and the implanted fibers were connected to a laser generator (Shanghai Fiblaser) using optical fiber sleeves. The delivery of blue light (473 nm, 1–3 mW, 15 ms pulses, 20 Hz) or yellow light (594 nm, 5–8 mW, constant) was controlled by a Master-8 pulse stimulator (A.M.P.I.). The same protocol was also used for the control group. At the end of the experiment, fiber location was evaluated and data obtained from mice with fibers located outside the expected brain region were discarded.

### Local drug infusion

A guide cannula (internal diameter 0.34 mm, RWD) was implanted toward the BLA and secured to the mice’s skull with dental cement. After the mice had recovered for at least 1 week, 200 nl of AP5 (2 mM, Sigma-Aldrich) and DNQX (2 mM, Sigma-Aldrich), dissolved in artificial cerebrospinal fluid (ACSF), were delivered through an internal stainless-steel injector attached to a 10 μl syringe (Hamilton) and an infusion pump (200 nl/min). The same volume of ACSF was injected into the controls. Two minutes after the infusion, the injector was slowly withdrawn and the behavioral assays were performed about 30 min later. The data obtained from the mice with missed injections were excluded from further analysis.

### RTPP/RTPA test

The mice were placed into the middle arena of the custom-made chamber, composed of two chambers (40 × 20 cm) connected by a “neck” structure, and allowed to freely explore the apparatus for 15 min. An observer recorded the time spent in each chamber to determine the baseline place preference or avoidance. The preference or avoidance chamber was defined as the “stimulation” side in RTPA or RTPP test during the 15 min testing period. During the 15 min test, laser stimulation was delivered whenever the mouse entered the center of the “stimulation” chamber and terminated once the mice left the chamber. The behavior of each testing mouse was recorded using a video camera and subsequently analyzed using EthoVision XT software (https://www.noldus.com/ethovision-xt). The preference or avoidance ratio was calculated as time spent in the stimulated side/baseline.

### Conditioned place avoidance test

For the light-induced conditioned place aversion, the mice were allowed to freely explore the apparatus for 15 min on the first day without any stimulation. Then, the light was delivered in the stimulation chamber on days 2, 4, and 6 and allowed to explore another chamber without light delivery on days 3, 5, and 7. On the eighth day, the mice were allowed to freely explore the apparatus without light delivery. The avoidance ratio for the stimulation chamber was calculated as time spent on the eighth day/first day.

### Brain slice electrophysiology

The acute brain slices used for whole-cell recording were prepared as previously described (52). Mice were deeply anesthetized and intracardially perfused with almost 20 ml of ice-cold oxygenated modified N-methyl-d-glucosamine (NMDG) ACSF, containing 93 mM of NMDG, 1.2 mM of NaH_2_PO_4_, 2.5 mM of KCl, 20 mM of N-2-hydroxyethylpiperazine-N-2-ethanesulfonic acid (Hepes), 30 mM of NaHCO_3_, 2 mM of thiourea, 25 mM of glucose, 5 mM of sodium ascorbate, 0.5 mM of CaCl_2_, 3 mM of sodium pyruvate, 10 mM of MgSO_4_, and 3 mM of glutathione. The pH was adjusted to 7.3 to 7.4 and osmolarity to 300 to 305 mmol/kg. The brain was quickly removed and coronal slices (300 μm), including the BLA, IC, and MD, were sectioned using a vibrating microtome (VT1200S, Leica). The slices were incubated in NMDG ACSF for 10 to 12 min at 33 °C and transferred to the oxygenated Hepes ACSF solution (28 °C), containing 20 mM of Hepes, 92 mM of NaCl, 2 mM of CaCl_2_, 25 mM of glucose, 30 mM of NaHCO_3_, 2.5 mM of KCl, 1.2 mM of NaH_2_PO_4_, 2 mM of thiourea, 5 mM of sodium ascorbate, 3 mM of sodium pyruvate, 3 mM of glutathione, and 2 mM of MgSO_4_ (pH 7.3–7.4, osmolarity of 300–305 mOsm/kg) for at least 1 h. Finally, the coronal slices were transferred to the recording chamber (Warner Instruments), which was continuously perfused with standard ACSF that contained 124 mM of CaCl_2_, 2.4 mM of NaCl, 5 mM of KCl, 26.2 mM of NaHCO_3_, 1.3 mM of MgSO_4_, 1.2 mM of KH_2_PO_4_, and 10 mM of glucose (pH: 7.3–7.4 osmolarity: 300–310 mOsm/kg) for whole-cell recording at 32 °C and 2.5 to 3 ml/min. The temperature of the ACSF during recording was maintained by an in-line solution heater (TC-344B, Warner Instruments), and the recorders were blinded to the group identity during recording and analyses.

Neurons in the BLA, IC, and MD were visualized using a 40 × water-immersion objective on an upright microscope (BX51WI, Olympus), equipped with IR-differential interference contrast, and identified *via* fluorescence, if applicable.

The recording pipettes were pulled from the borosilicate glass capillaries (VitalSense Scientific Instruments Co, Ltd) with an outer diameter of 1.5 mm on a four-stage horizontal puller (P1000, Sutter Instruments) to a tip resistance of 3 to 5 MΩ when filled with intracellular solution that contained 2 mM of MgCl_2_, 5 mM of KCl, 130 mM of K-gluconate, 10 mM of Hepes, 0.6 mM of EGTA, 2 mM of Mg-ATP, and 0.3 mM of Na-GTP (osmolarity: 285–290 mOsm/kg, pH: 7.2). The signals were acquired using Multiclamp 700B amplifier (2 kHz low-pass filter, 10 kHz digitization) and a Digidata 1440A digitizer (Axon Instruments). The recording was immediately terminated once the series resistance changed more than 20% during the recording. For recording the activity of BLA neurons, current-evoked firing was recorded in current-clamp mode (*I* = 0 pA) using pipettes filled with intracellular solution. The threshold current for firing was defined as the minimum current required to elicit an action potential. For recording the current-evoked firing of BLA-projecting IC or MD neurons, the alexafluor 488-conjugated cholera toxin subunit B (CTB-488, Thermo Fisher Scientific) was injected into the BLA, and the CTB-488–positive neurons in the IC or MD were recorded. For the miniature excitatory postsynaptic current recordings, the neurons were held at −70 mV and the picrotoxin (40 μM, Sigma-Aldrich) and tetrodotoxin (100 nM) were added into the standard ACSF. For the light-evoked response, neurons were held at −70 mV using the voltage clamp mode and the light (473 nm for activation or 594 nm for inhibition) was delivered using a laser (Shanghai Fiblaser Technology Co, Ltd) through an optical fiber 200 μm in diameter, positioned 0.2 mm above the surface of interested areas. The recorded data were analyzed offline using Clampfit 10.7 software (Molecular Devices). Unless otherwise stated, all reagents were purchased from Sigma-Aldrich. Tetrodotoxin was obtained from Hebei Aquatic Science and Technology Development Company.

### Statistical analysis

We conducted simple statistical comparisons using Student’s *t* test. For experimental groups with multiple comparisons, ANOVA (one-way or two-way) with *posthoc* analyses were used to statistically analyze the data. GraphPad Prism 9 (https://www.graphpad.com/) was used for the statistical analyses and graphing. Clampfit software 10.7 and MiniAnalysis software (https://synaptosoft.software.informer.com/) were used to conduct offline analyses of electrophysiological data. All data are expressed as the mean ± SEM, and significance levels are set as ∗*p* < 0.05, ∗∗*p* < 0.01, ∗∗∗*p* < 0.001.

## Data availability

The data that support the findings of this study are all contained in the results section of the article and also available upon request. For requests, please contact Wenjie Zhou at zwj2850@ustc.edu.cn.

## Supporting information

This article contains supporting information

## Conflict of interest

All authors report no biomedical financial interests or potential conflicts of interest. The authors declare that they have no conflicts of interest with the contents of this article.
