# Supplementary material for: Distinct basolateral amygdala excitatory inputs mediate the somatosensory and aversive-affective components of pain
Source: J Biol Chem. 2022 Jun 27;298(8):102207. doi: 10.1016/j.jbc.2022.102207 (PMC9304789; doi:10.1016/j.jbc.2022.102207)
Supplement: Supporting information [file mmc1.docx]

**Supporting Information for**

**Distinct basolateral amygdala excitatory inputs mediate the somatosensory and aversive-affective components of pain**

Xiaojing Meng^1†^, Lingxiao Yue^2,3†^, An Liu^2†^, Wenjuan Tao^2^, Li Shi^1^, Wan Zhao^4^, Zhongmin Wu^5^, Zhi Zhang^6^, Liecheng Wang^2^*, Xulai Zhang^1^* and Wenjie Zhou^6^*

^1^Affiliated Psychological Hospital of Anhui Medical University, Hefei Fourth People’s Hospital, Anhui Mental Health Center, Hefei 230022, China

^2^Department of Physiology, School of Basic Medical Sciences, Anhui Medical University, Hefei 230022, China

^3^Department of Pathology, Anhui Medical College, Hefei 230601, China

^4^Department of Otolaryngology, The First Affiliated Hospital of USTC, Division of Life Sciences and Medicine, University of Science and Technology of China, Hefei, Anhui, 230001, China.

^5^Department of Anatomy, Medical College of Taizhou University, Taizhou 318000, China.

^6^Hefei National Laboratory for Physical Sciences at the Microscale, CAS Key laboratory of Brain Function and Disease, University of Science and Technology of China, Hefei 230027, China.

^6^These authors contributed equally: Xiaojing Meng, Lingxiao Yue, An Liu

*Correspondence: Wenjie Zhou

E-mail: [zwj2850@ustc.edu.cn](mailto:zwj2850@ustc.edu.cn);

Xulai Zhang

xulaizhang@ahmhcentre.com;

Liecheng Wang

E-mail: wangliecheng@ahmu.edu.cn.

**Figure S1.** Inputs of BLA^Glu^ neurons.

(**A**) Schematic of viral injection in the ACx of *CaMKII-Cre* mice. (**B** to **E**) Representative images of EGFP signals in the indicated regions. CeM, central amygdala nucleus, medial division; Pir, piriform cortex; ACx, auditory cortex; Ect, ectorhinal cortex; PO, posterior thalamic nuclear; PF, parafascicular thalamic nucleus. Scale bars, 500 μm.
