# Supplementary figures and images for: Distinct basolateral amygdala excitatory inputs mediate the somatosensory and aversive-affective components of pain
Source: J Biol Chem. 2022 Jun 27;298(8):102207. doi: 10.1016/j.jbc.2022.102207 (PMC9304789; doi:10.1016/j.jbc.2022.102207)

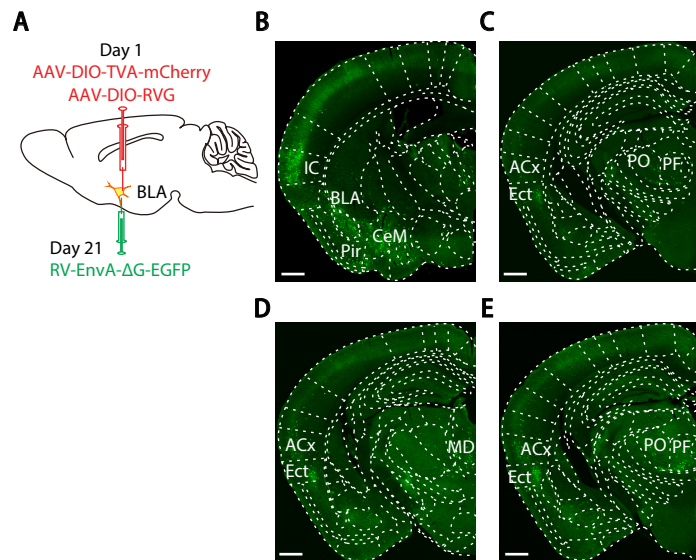

Supplement: Figure S1 [file mmc2.pdf]
